# Investigation of the coupling quality of partial prostheses at the stapes head

**DOI:** 10.1007/s00405-024-09105-x

**Published:** 2024-11-29

**Authors:** Sijia Zhai, Till Moritz Eßinger, Martin Koch, Jianhang Deng, Thomas Zahnert, Marcus Neudert, Matthias Bornitz

**Affiliations:** Carl Gustav Carus Faculty of Medicine, Department of Otorhinolaryngology, Head and Neck Surgery, Ear Research Center Dresden, Technische Universität Dresden, Fetscherstraße 74, 01307 Dresden, Germany

**Keywords:** Middle ear reconstruction, Partial ossicular replacement prosthesis (PORP), Coupling, Total harmony distortion (THD), Laser doppler vibrometer (LDV)

## Abstract

**Introduction:**

The success of partial ossicular chain reconstructions in cases of conductive hearing loss depends on many factors. One important factor is the coupling between the prosthesis and the stapes head, which has not been explicitly investigated previously. Prostheses with different forms of attachment to the stapes are available, namely clip and bell type PORP. We present a standardized method to assess the quality of the prosthesis-stapes connection. The coupling quality of different prostheses is compared using measurements on a specimen model.

**Methods:**

This study delineated six groups categorized by prosthesis types, employing 12 temporal bones to create a reconstructed ossicular chain model. The model comprised stapes and inner ear of the specimen, various prostheses and a standardized mechanical excitation at the prosthesis head. Multiple-points measurements were conducted using laser Doppler vibrometry along the sound transfer direction. This methodology enabled the assessment of vibrational magnitude loss and sound distortion from the prosthesis to the stapes.

**Results:**

All six groups showed uniformly good sound transmission, with low magnitude loss of < 10 dB and very low total harmonic distortion of < 1%.

**Conclusion:**

The proposed measurement method enables an explicit and comparable examination of the prosthesis coupling to the stapes head. While the coupling mechanism may be important in terms of handling, stability or long-term robustness of the reconstruction, our results show no relevant differences between types in terms of sound transmission.

**Supplementary Information:**

The online version contains supplementary material available at 10.1007/s00405-024-09105-x.

## Introduction

Conductive hearing loss, resulting from the impairment of sound transmission by air conduction from the external environment through the middle ear to the inner ear, is often repaired through partial ossicular chain reconstruction, when the residual ossicular chain is still available [1]. Various prostheses have been previously developed to address ossicular chain reconstruction, with clip and bell type partial ossicular replacement prostheses (PORP) being prominent solutions [2, 3].

The result of such reconstruction depends upon multiple factors. The postoperative pathological variations between patients (especially ventilation and scarring) play a dominate role in the audiological outcome [4–6]. The mechanical properties of prostheses (mass, rigidity, prosthesis head size etc.) have effects on vibration transmission [7]. Increased tension of the reconstructed ossicular chain is often necessary for the stability of reconstruction and also functions to restrict unwanted prosthesis rotation, but it significantly reduces sound transmission at lower frequencies below 1 kHz, underlining the importance of balance between preservation of mechanical vibrations and optimal stability [8]. The handling characteristics of prosthesis during surgery, being highly relevant to surgeons’ subjective preference and technique, is also an important factor [9].

For PORP, simulations show that the coupling quality between the prosthesis and the stapes head is one crucial factor affecting the success of the reconstruction [10]. A secure and stable coupling can lead to improved acoustic transfer, while poor coupling results in sound conductive hearing loss and/or distortions in sound perception, leading to suboptimal hearing restoration. The coupling condition of implants can be determined by coupling mechanism of each prosthesis type, namely different forms of attachment to stapes, such as clip or bell type. Various mechanisms may result in varying degrees of sound vibration loss and distortion across the coupling interfaces, which is often due to gap existing or excessive rotational movements replacing efficient piston-like motions at this interface. Experimental investigation on stapesplasty also show, that imperfect coupling could result in relative motion between ossicles and prosthesis [11].

Despite the recognized importance of prosthesis-stapes coupling, there has been little explicit investigation evaluating the coupling qualities under various attachment mechanisms. Therefore, we propose a novel, standardized method to evaluate the prosthesis-stapes coupling in terms of sound transmission and quality. The sound quantity changes were measured through vibration magnitude loss at the coupling interface. While total Harmonic Distortion (THD), which reflects sound distortion through the presence of harmonic frequencies in the output, was used to quantify sound quality changes.

Stapes footplate motion is composed of different modes of vibration, and especially in reconstructed middle ears, rotational motions need to be considered [12, 13]. However, the majority of previous studies used single point method in laser Doppler Vibrometry (LDV) measurements, where results may vary with the exact measurement position [14]. We therefore used both single point and five-point methods for LDV measurements and compared the two methods.

## Methods and materials

The entire study was conducted in compliance with the Declaration of Helsinki and the principles of good scientific practice.

### Preparation

Twelve freshly frozen human cadaveric temporal bones (TBs) were prepared for this study. Before freezing, a general evaluation of TBs was performed to ensure the integrity of middle ear and tympanic membrane (TM) by observation with microscopy. They were then thawed in water at 4 °C for 24 h and warmed to room temperature before the measurement. Mastoid approach and posterior tympanotomy were used to reach the middle ear cavity. The facial recess was opened to expose the stapes footplate [15].

After finishing the LDV measurements for the intact TBs, the incus was carefully removed through tympanic antrum, and necessitating the severance of both the incudostapedial joint (ISJ) and the superior ligament of the incus in advance. A saw was then employed to excise the structures (including partial ossicular chain) external to stapes, only preserving the stapes and the following inner ear structures. Throughout the process, utmost care was taken to maintain the stapes integrity, especially footplate as well as the annular ligament.

### Experimental protocol

**Fig. 1:**
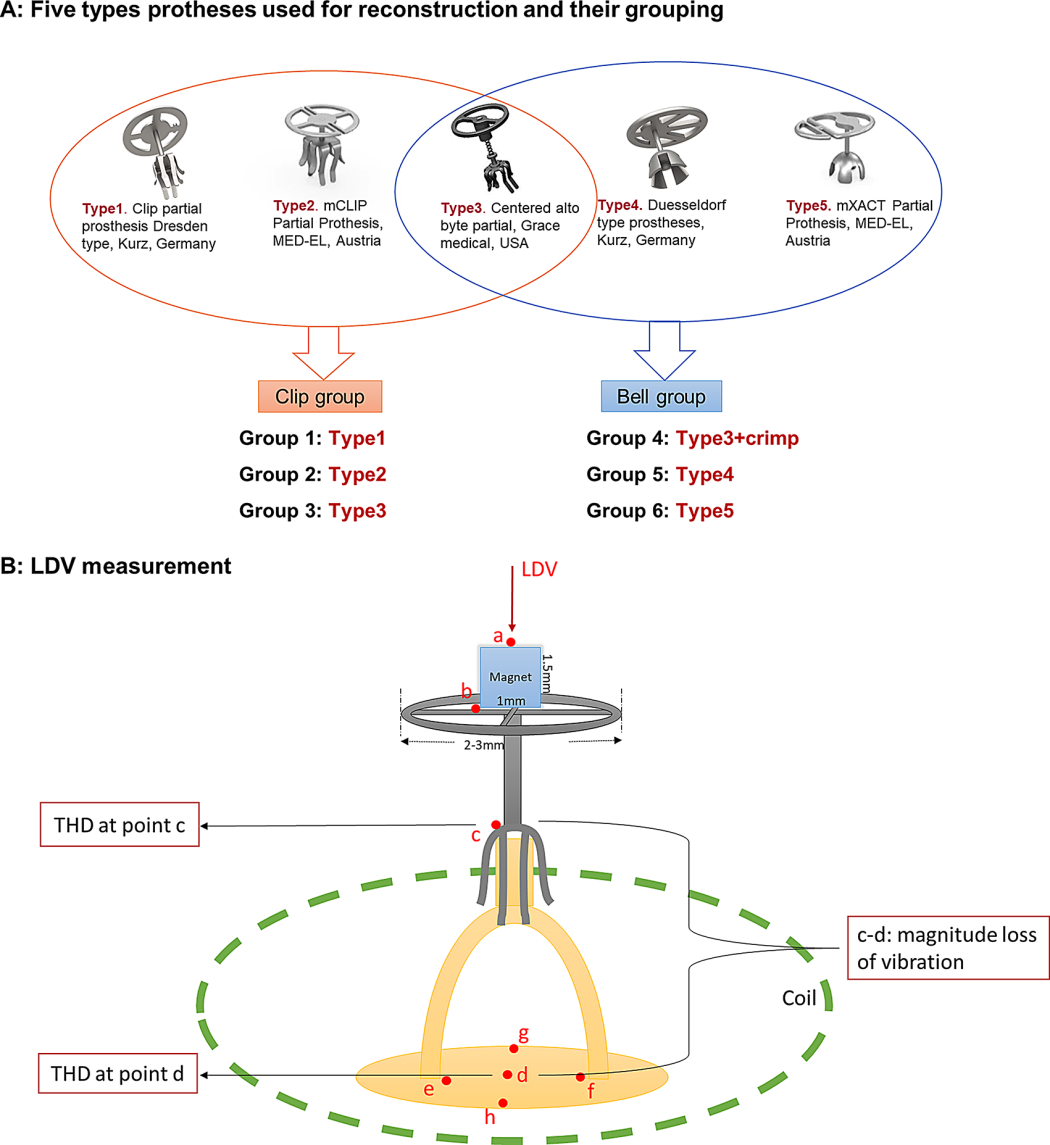
**A**: Five Prostheses utilized in this study: Type1: Clip partial prosthesis Dresden type, Kurz, Germany. Type2: mCLIP Partial Prothesis, MED-EL, Austria. Type3: Centered alto byte partial, Grace medical, USA. Type4: Duesseldorf type prostheses, Kurz, Germany. Type5: mXACT Partial Prothesis, MED-EL, Austria. Type 3 was coupled using two methods, resulting in six groups from five prosthesis types. **B**: Schematic diagram of LDV measurement for each reconstruction with various prostheses, red points indicate the targets measured by laser (target a: magnet, b: prosthesis plate, c: prosthesis foot, d: stapes footplate center, e: stapes posterior foot, f: stapes anterior foot, g: middle point of lateral side of footplate, h: middle point of medial side of footplate)

The prepared specimens were coupled with various prosthesis types. Using wax, a magnet was attached to the prosthesis top. The specimen was placed inside a coil, so that magnet and coil served as the actuator (see Fig. 1B). This study involves five types of prostheses: two clip prostheses that attach to the stapes with spring-like elements (Type 1: Clip Partial Prosthesis Dresden, Kurz, Germany; Type 2: mCLIP Partial Prosthesis, MED-EL, Austria), two bell prostheses that require additional crimping procedure (Type 4: Duesseldorf Type Prostheses, Kurz, Germany; Type 5: mXACT Partial Prosthesis, MED-EL, Austria), and one clip-shaped prosthesis (Type 3: Centered Alto Byte Partial, Grace Medical, USA). The Type 3 prosthesis does not provide real spring-like element but its clip shape has larger contact area compared to the bell type. It can be used in two ways: loosely placed at the stapes head or tightly crimped like a bell. Therefore, six groups were created with the five prosthesis types in this study (see Fig. 1A).

LDV measurements were taken at multiple points a-d (a: magnet, b: prosthesis plate, c: prosthesis foot, d: stapes footplate center), along the direction of sound transmission, allowing for the assessment of vibrational magnitude loss and total harmonic distortion (THD). To minimize the bias caused by rotational motions, all the measuring points a-d were selected as close as possible to the axis perpendicular to the footplate center (see Fig. 1B). To confirm the stability of the magnet on the prosthesis, the vibrations curves at points a and b were checked. Vibrational magnitude loss was obtained by subtracting the magnitude at point d from that at point c. THD was calculated as the ratio of harmonic sums up to the sixth harmonic at points c and d (across the coupling interface). All above data were utilized for comparative analysis among prosthesis groups.

Stapes footplate (SFP) motion was evaluated by analyzing vibrational responses at five points on the footplate (d: center point, f: anterior foot, e: posterior foot, h: middle point of medial side, g: middle point of lateral side), with the coordinates of these points determined using ImageJ software (National Institutes of Health, Bethesda, MD, USA) from the photos taken during measurements. At the end, the translational velocity calculated via the 5-point method was compared with the center point velocity of SFP obtained by single-point method for validation.

### LDV measurements

Initially, the middle ear transfer functions (METFs) of each intact TB were measured and compared with standard ranges in the literature to exclude functionally abnormal specimen that exhibit pathological sound transmission of the intact structure [16]. The measurement setup comprised an earphone (EARtone 3 A) for sound stimulation and a probe microphone (ER7c, both Ethymotic Research, Inc., Elk Grove Village, Illinois, U.S.A.) for measuring the input sound pressure to the TM. An approx. 0.05 mm^2^ reflective foil was placed on the footplate center to enhance signal capture for the movement measurement by LDV (laser head CLV 700, controller CLV 1000, Polytec PI, Waldbronn, Germany). The LDV was mounted to a micro manipulator (A-HLV-MM40, Polytec PI, Waldbronn, Germany) and both were mounted to an operating microscope (OPMI 111, Carl Zeiss, Oberkochen, Germany).

**Fig. 2:**
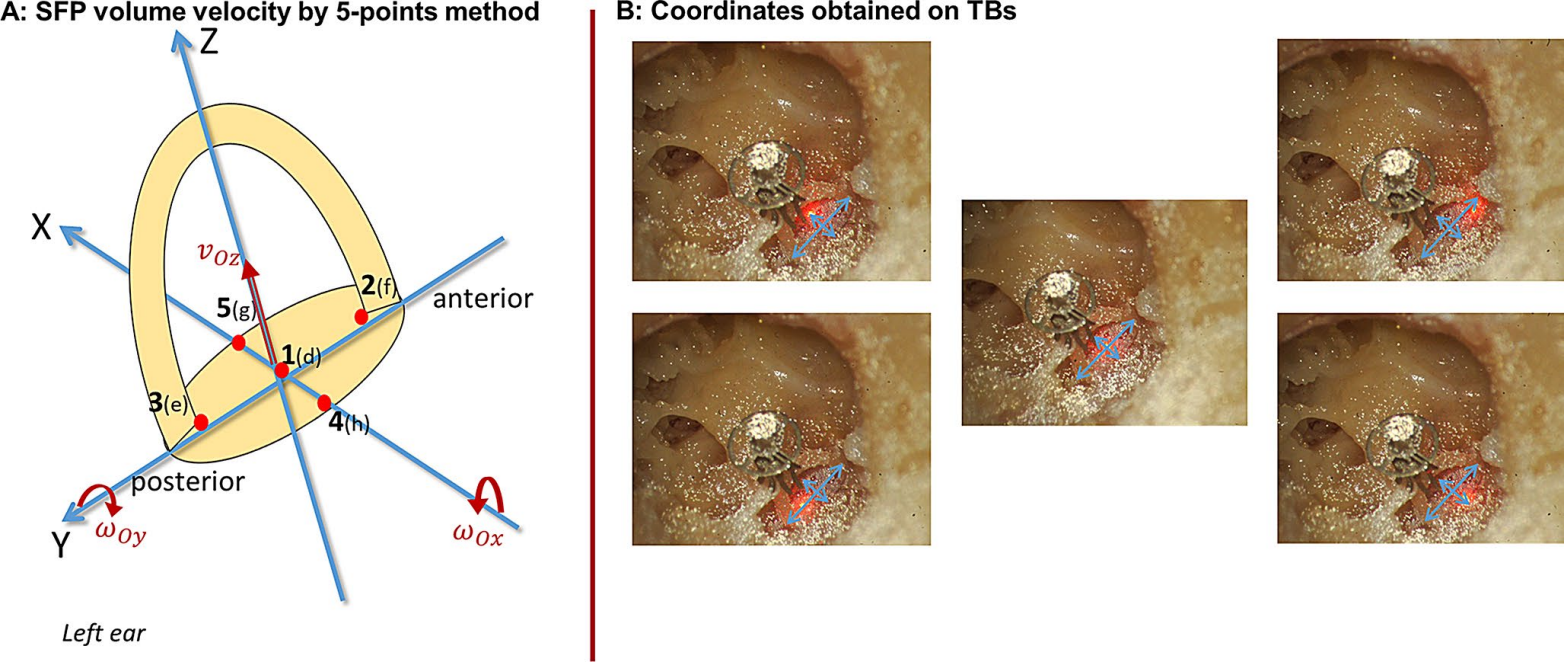
**A**: Rigid body motions of the stapes at its footplate center. The relevant components are marked red at the axes, the translational velocity $$\:{v}_{Oz}$$ and the rotational component $$\:{\omega\:}_{Oy}$$ and $$\:{\omega\:}_{Ox}$$. They are derived from the data $$\:{v}_{1z}$$, $$\:{v}_{2z}$$$$\:{v}_{3z}$$$$\:{v}_{4z}$$ and $$\:{v}_{5z}$$ measured at targets 1(d), 2(f), 3(e), 4(h) and 5(g). **B**: The photos of one TB during one five-point measurement process, on which coordination axes are drawn in batch. The scale for pixel distances were respectively set to 2.83 mm for the long axis and 1.3 mm for the short axis within the photographs. The coordinates of the five designated points were measured from these images

Subsequently, ossicular reconstructions were performed on stapes under a fully exposed vision after the removal of partial structures. The application of the prostheses was randomized to prevent any order-based experimental bias. Reflective powders were placed on the area of measurement targets at the prosthesis after successful reconstruction. A laser Doppler vibrometer LDV (VibroOne, Polytec PI, Waldbronn, Germany), was utilized to realize the measurements after every reconstruction. The LDV was mounted to a micromanipulator (as above) and microscope (OPMI MDM, Carl Zeiss, Oberkochen, Germany). During the process, the laser beam of the LDV (spot size 0.1 mm^2^ ) was positioned with the micromanipulator at the various points one by one, as shown in Fig. 2, to measure the vibration and THD. Excitation was performed with the coil-magnet system as described before; the excitation level is 85 ± 15 dB SPL (mean ± SD).

### Calculation of SFP motion by 5-point method

In our study a multiple point method as stated by Hato et al. was taken to caculate the rigid body motion of the SFP [13]. The motion of the stapes as a rigid body can be described by three translations and three rotations at the stapes footplate center (see Fig. 2A). Among them only the components relevant to the sound transmission mechanics ($\:{v}_{Oz},\:{\omega\:}_{Ox},\:{\omega\:}_{Oy}$) were taken into the following calculation. The motion at any measurement point P(x, y) on the SFP can be calculated using the formula

$$\:{v}_{pz}\:\left({x}_{p}{,\:y}_{p}\right)=\:{v}_{Oz}+{\omega\:}_{Ox}\:\text{*}\:{y}_{p}-\:{\omega\:}_{Oy}\:\text{*}\:{x}_{p}$$

With LDV we measured motions in z-direction at five points on the footplate, obtaining $\:{v}_{1z}$- $\:{v}_{5z}$ (see Fig. 2A). With these data we get an over determined system of equations for the translation $\:{v}_{Oz}$ and rotation $\:{\omega\:}_{Ox}$ and $\:{\omega\:}_{Oy}$:

$$\:\left[\begin{array}{c}{v}_{1z}\\\:{v}_{2z}\\\:{v}_{3z}\\\:{v}_{4z}\\\:{v}_{5z}\end{array}\right]=\left[\begin{array}{ccc}1&\:{y}_{1}&\:-{x}_{1}\\\:1&\:{y}_{2}&\:-{x}_{2}\\\:1&\:{y}_{3}&\:-{x}_{3}\\\:1&\:{y}_{4}&\:-{x}_{4}\\\:1&\:{y}_{5}&\:-{x}_{5}\end{array}\right]\:\left[\begin{array}{c}{v}_{Oz}\\\:{\omega\:}_{Ox}\\\:{\omega\:}_{Oy}\end{array}\right]\:\text{o}\text{r}\:\left[\begin{array}{c}{v}_{1z}\\\:{v}_{2z}\\\:{v}_{3z}\\\:{v}_{4z}\\\:{v}_{5z}\end{array}\right]=X\:\left[\begin{array}{c}{v}_{Oz}\\\:{\omega\:}_{Ox}\\\:{\omega\:}_{Oy}\end{array}\right]$$

This system can be solved with a minimum least square approach:

$$\:\left[\begin{array}{c}{v}_{Oz}\\\:{\omega\:}_{Ox}\\\:{\omega\:}_{Oy}\end{array}\right]={\left({X}^{T}X\right)}^{-1}{X}^{T}\left[\begin{array}{c}{v}_{1z}\\\:{v}_{2z}\\\:{v}_{3z}\\\:{v}_{4z}\\\:{v}_{5z}\end{array}\right]$$

The coordinates $\:\left({x}_{p}{,\:y}_{p}\right)\:$of the measurement points were obtained from photographs using software ImageJ, as shown in Fig. 2B. The mean footplate length of 2.83 mm and width of 1.3 mm served as reference dimensions for all specimens. To realize the comparison between five-point method and single point method, the difference of the rigid body translational velocity of the stapes $\:{v}_{Oz}$ and the measured velocity at point d,$\:\:{v}_{1z}$ was calculated.

### Statistical analysis

Given that THD is easily affected by the environmental noise, THD data with a signal to noise ratio of less than 20 dB were excluded from the dataset before conducting statistical analysis.

The data sets were categorized into six frequency bands. Within each band, averaging was performed using equal subdivisions on a logarithmic scale to represent the band as a whole. For instance, data ranging from 355 to 707 Hz were averaged to represent the 500 Hz band. The audiological frequency bands, specifically 250, 500, 1000, 2000, 4000, and 8000 Hz bands, were used in the analysis (see Table S1).

After assessing the data distribution normality and variance homogeneity for all magnitude and THD data, a comparative analysis was conducted on magnitude loss between point c and d and on THD at point c and d across the six groups categorized by the prosthesis types, using the Analysis of Variance (ANOVA) method. If the ANOVA result was significant, Tukey-HSD tests were performed for pairwise comparisons. Furthermore, to more thoroughly investigate the THD change across the coupling interface, the difference in THD between points c and d was assessed among the six groups using an independent t-test. Lastly, the difference of the rigid body translational velocity of the stapes $\:{v}_{Oz}$ and the measured velocity at point d, $\:{\widehat{v}}_{1z}$ was compared between groups using a paired t-test after verifying the normality of the data. All the statistical analyses were performed by SPSS 29.0 software.

## Results

### Sound quantity − magnitude loss of vibration during sound transmission through the coupler interface

**Fig. 3:**
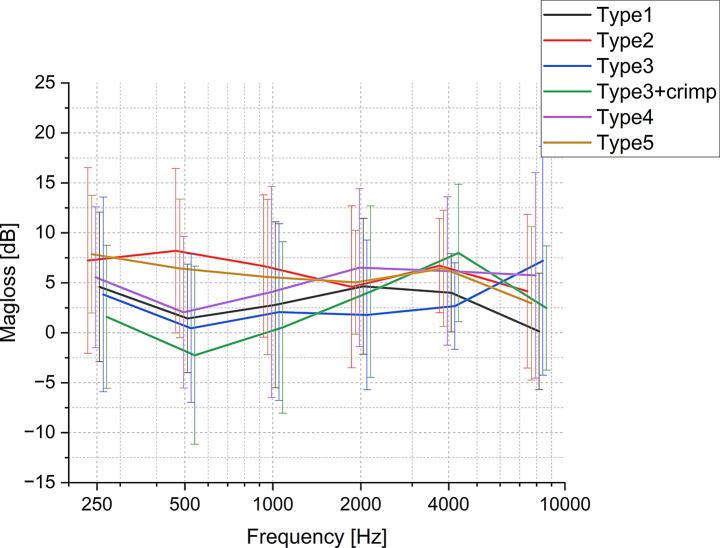
Mean magnitude loss and SD. across the coupling interface (cp. Figure 1B) at a range of frequencies (from 200 Hz to 10,000 Hz, with offset between groups along x-axis) for various types of clips and bells. (*n* = 12)

As illustrated in Fig. 3 magnitude loss fluctuates with frequency, exhibiting greater variance among all prostheses types at lower frequencies (< 1000 Hz) compared to higher frequency (≥ 1000 Hz). All prosthesis types, except Type2 and Type5, show a general increase in mean magnitude loss from 500 to 4000 Hz. Above 4000 Hz, most groups show a decrease, but Type3 exhibits an increase. Overall, the mean magnitude losses for all prosthesis types range no more than 10 dB, but with relatively larger standard deviations (SD).

**Fig. 4:**
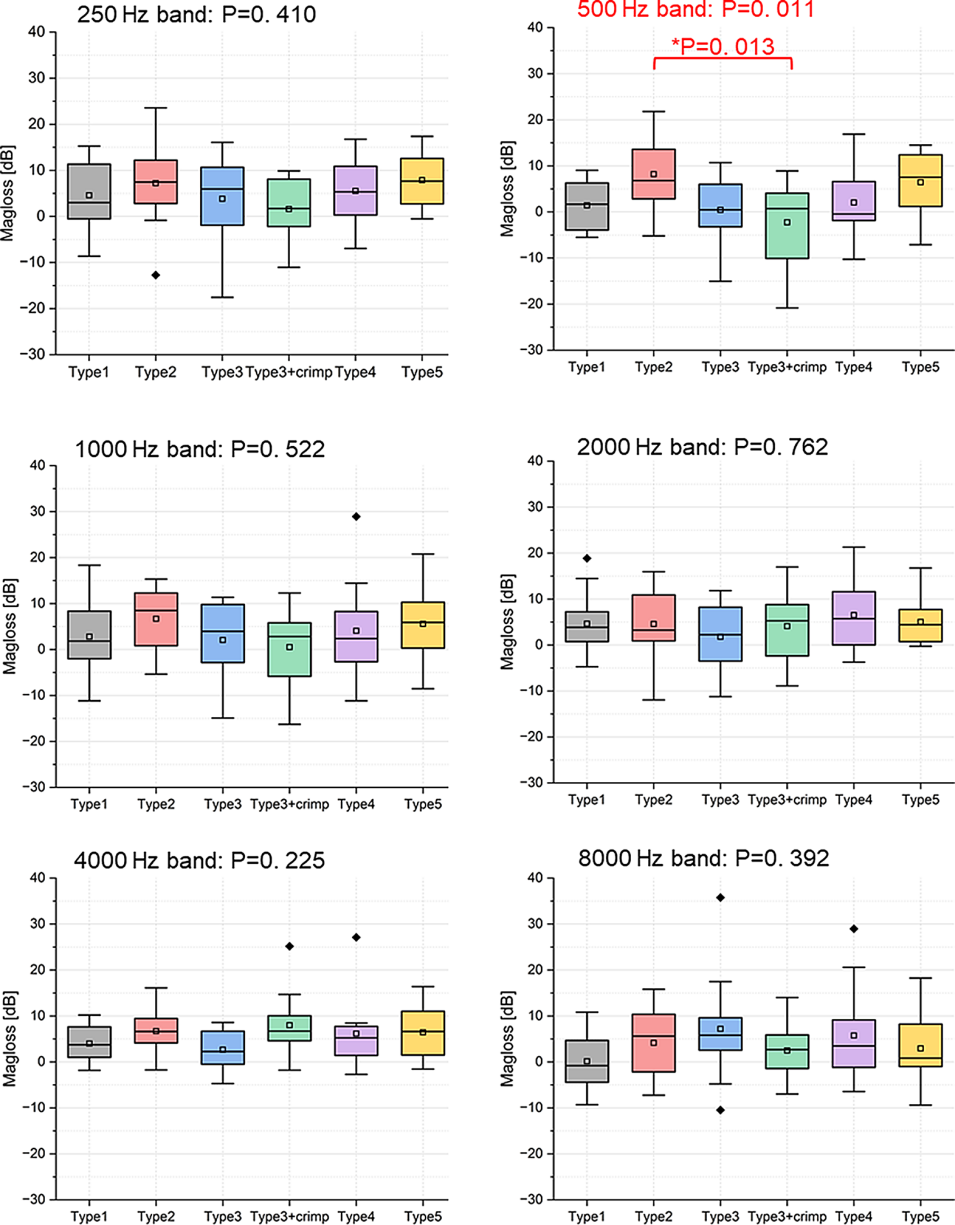
The effect of prosthesis type on magnitude loss at each frequency band. (*n* = 12) Boxes show: mean, median, interquartile ranges, whisker with coef of 1.5, outliers

The differences in magnitude loss through the coupler interface between groups are not significant, except for Type2 and Type3 + crimp within the 0.3–0.7 kHz band (Fig. 4), suggesting that prosthesis type has almost no effect on the vibration magnitude at each frequency band.

### Sound quality– THD on the reconstructed ossicular chain

**Fig. 5:**
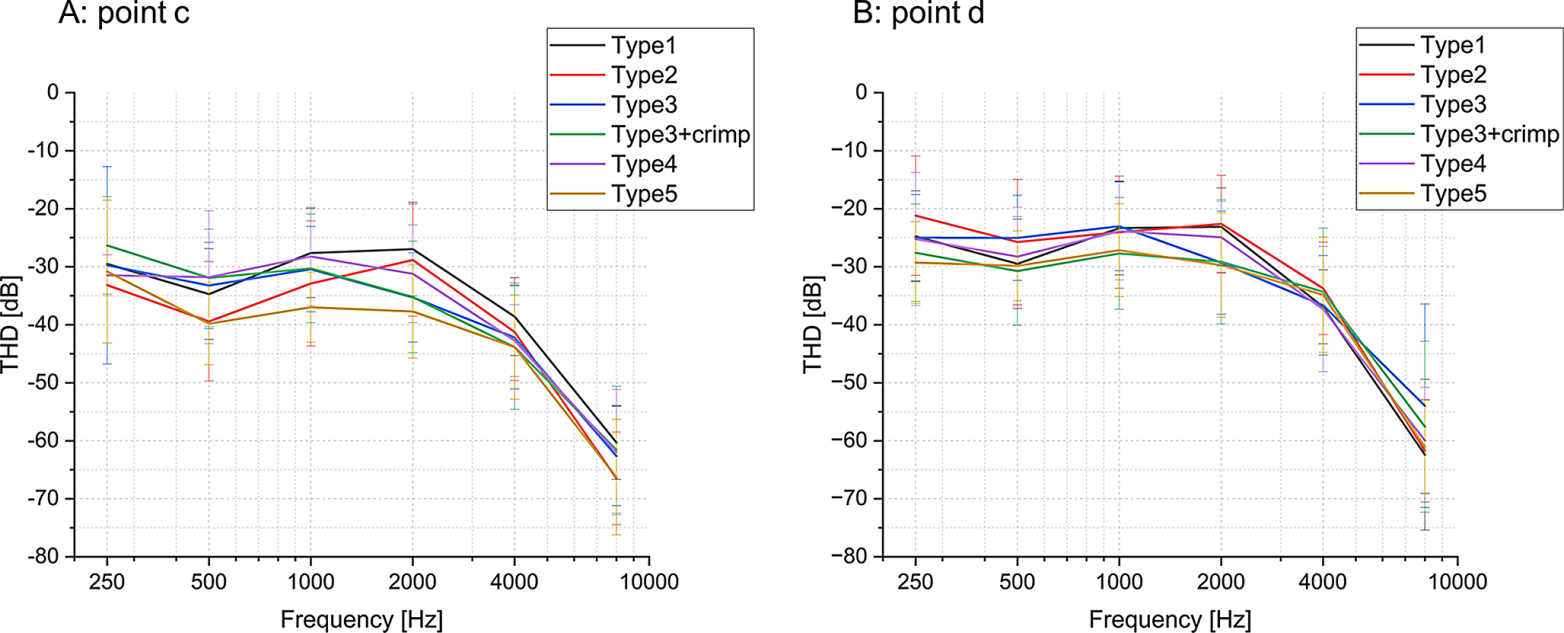
The means and SD of THD values (*n* = 12) for various prostheses types labeled as Type1, Type2, Type3, Type4, Type5 and Type3 + crimp across a frequency range of 200 Hz to 10,000 Hz. A: the THD values at point c. B: the THD at point d

**Fig. 6:**
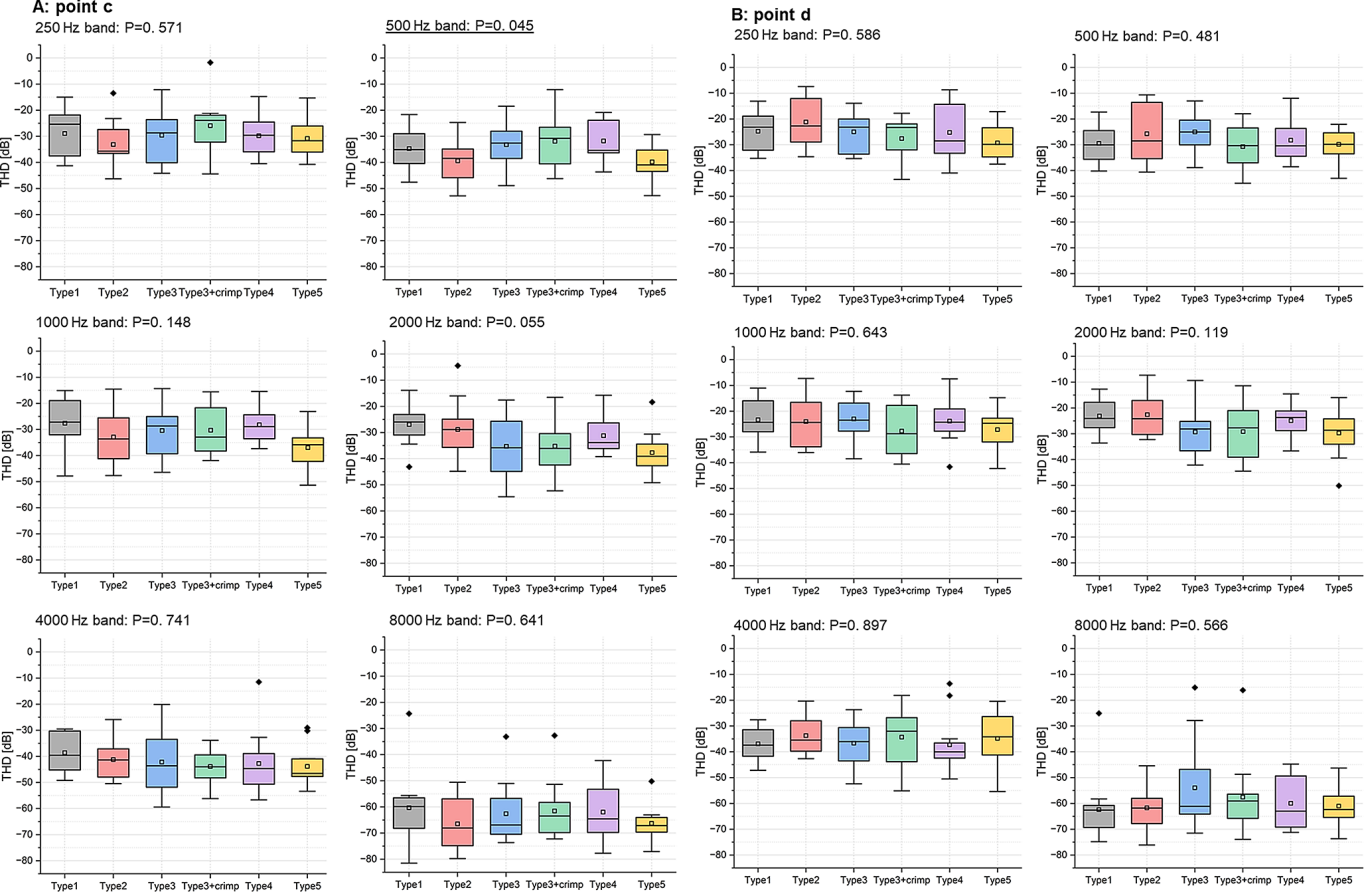
Box plots for the THD values for each prosthesis type (*n* = 12) at **A**: point c and **B**: point d. Boxes show: mean, median, interquartile ranges, whisker with coef of 1.5, outliers. The underlining P value shows a significant ANOVA test at 500 Hz, but no significance in subsequent Tukey-HSD test

THD values at point d (Fig. 5b) are generally higher than those at point c (Fig. 5a) and exhibit a peak at about 1000–2000 Hz, dropping off towards higher frequencies. Statistical analysis reveals no significant difference between prosthesis types at either point (Fig. 6 and Table S2, S3, S4). However, independent t-tests show that the THD difference (THD at point c– THD at point d) is significant in some cases (see Table 1). Type1 does not exhibit significant THD differences across all frequencies, indicating minimal sound distortion with this type. Conversely, Type3, Type3 + crimp, and Type4 exhibited significant THD variations at certain frequency bands. Furthermore, Type2 and Type5 showed the most significant THD differences among all the groups.

**Table 1:** Change in total harmonic distortion at coupling interface (between points c and d) for prosthesis types examined. Significant values are marked in red color

Group	Band Frequency (k Hz)	0.25 (0.177–0.354)	0.5 (0.355–0.707)	1 (0.708–1.414)	2 (1.414–2.828)	4 (2.829–5.657)	8 (5.658-10.000)
Type1	difference	-4,186	-5,225	-4,289	-3,85392	-1,66248	2,10283
	P value	0,327	0,128	0,249	0,205	0,577	0,704
Type2	difference	-11,967	-13,689	-8,848	-6,23388	-7,50244	-4,75271
	P value	0,015*	0,002*	0,038*	0,134	0,028*	0,228
Type3	difference	-4,616	-8,198	-7,405	-5,99475	-5,53371	-8,66294
	P value	0,220	0,018*	0,062	0,172	0,219	0,169
Type3 + crimp	difference	1,650	-1,187	-2,572	-6,06773	-9,55339	-3,99401
	P value	0,706	0,763	0,515	0,182	0,015*	0,461
Type4	difference	-4,576	-3,558	-4,372	-6,30338	-5,43782	-2,01166
	P value	0,345	0,287	0,172	0,028*	0,256	0,634
Type5	difference	-1,544	-9,980	-9,807	-7,98731	-9,02743	-5,25235
	P value	0,654	<,001*	0,007*	0,032*	0,021*	0,088

### Measuring method comparison: 1-point vs. 5-point method

**Fig. 7:**
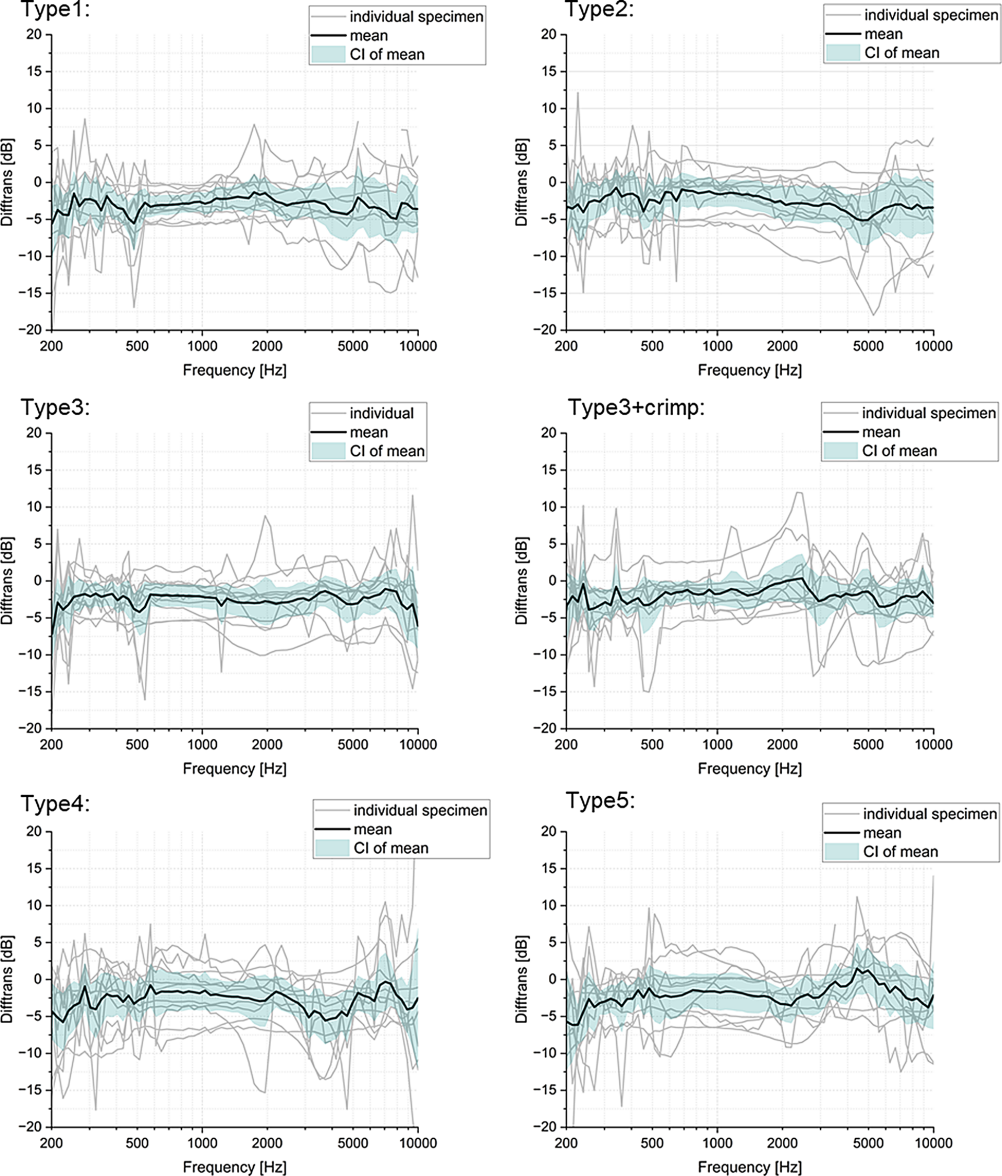
The difference between measurement at point d and calculated translation of the footplate (measurement methods 1-point vs. 5-points) across frequencies. The data for each prosthesis group were separately displayed with the label of prosthesis name. (*n* = 12)

Comparing the two measurement methods (1-point vs. 5-point LDV measurement) shows mean differences across the frequency bands of less than 5 dB magnitude (Fig. 7), with statistically significant differences for most measurement points and all prosthesis types (Table 2). Among clip groups, Type1 shows significant differences from 500 to 8000 Hz, Type2 at 250, 500, 2000, and 4000 Hz frequency bands, and Type3 across 250 to 8000 Hz. Among bell groups, Type3 + crimp shows significant differences at 250 and 2000 Hz frequency bands, Type4 at 250, 500, 4000, and 8000 Hz, and Type 5 at 250, 2000 and 4000 Hz.

**Table 2:** Difference between LDV measurement methods (1-point vs. 5 points) for each prosthesis type (*n* = 12)

Group	Band Frequency (k Hz)	0.25 (0.177–0.354)	0.5 (0.355–0.707)	1 (0.708–1.414)	2 (1.414–2.828)	4 (2.829–5.657)	8 (5.658-10.000)
Type1	difference	-2,400	-3,019	-2,604	-2,170	-3,016	-3,923
	P value	0,185	0,002*	<,001*	0,007*	0,020*	0,022*
Type2	difference	-3,570	-2,017	-1,423	-2,446	-3,837	-3,330
	P value	0,003*	0,032*	0,114	0,027*	0,006*	0,056
Type3	difference	-3,298	-2,456	-2,277	-2,885	-2,440	-3,683
	P value	<,001*	0,005*	0,006*	0,015*	0,016*	0,035*
Type3 + crimp	difference	-4,635	-2,649	-1,862	-2,904	-0,955	-1,398
	P value	0,001*	0,053	0,137	0,003*	0,331	0,332
Type4	difference	-3,260	-2,515	-1,544	-0,684	-2,007	-2,577
	P value	0,002*	0,017*	0,065	0,527	0,016*	0,018*
Type5	difference	-4,300	-2,096	-1,859	-2,464	-4,191	-2,293
	P value	0,003*	0,052	0,098	0,022*	0,001*	0,093

## Discussion

This study aimed to evaluate the coupling quality of different partial ossicular replacement prostheses (PORPs) to the stapes head using a standardized measurement method. Our findings reveal that while prosthesis-stapes coupling is critical for sound transmission, the variations in coupling mechanisms among different prosthesis types (clip and bell types) have minimal impact on overall acoustic performance.

### Proposed method for evaluating prosthesis-stapes coupling

Although the importance of prosthesis-stapes coupling is widely recognized, most studies use METF or hearing tests to evaluate overall reconstruction results, which are influenced by many factors. Our new method focuses specifically on prosthesis-stapes coupling to determine differences in sound transmission among prostheses. It assesses both sound quantity and quality by measuring signal loss and distortion at the coupling interface. This allows for a comparison of different coupling mechanisms, offering a valuable tool for prosthesis design. However, this method does not account for biological factors that could affect performance over time.

### Errors and limitations

1-point versus 5-point method: With the 1-point method, the rotations cannot be recorded. This means that these measurements increasingly deviate from the rigid body translational component if there is a large amount of rotation [12]. 5-point method on the other hand is strongly influenced by errors in coordinate measurements. This means that both methods can ultimately be equally error-prone.

Type 1 and Type 2 prostheses have actually identical clips (shape, material and material thickness). They are only from different manufacturers. Both clips are elastically widened when they are put on the stapes head and therefore fit snugly to the stapes in their final position. The expectation here was the same results for both types of prostheses. The differences may be explained by the different initial condition of the prostheses. The clips may have been spaced differently in the initial state, which meant that they did not fit as well with smaller stapes heads. The exact initial state of the clips was not recorded prior to the measurements. After using (i.e. elastic widening during coupling), the sizes of the clips are Type 1 (0.677 ± 0.075 * 1.045 ± 0.055) Type 2 (0.781 ± 0.039 * 1.011 ± 0.052).

Plastic deformation when fitting the clips should have affected both types equally and can therefore only be one explanation for the scattering of the results.

The measurement setup favors larger rotations of the stapes compared to the real reconstructed middle ear, as the coupling of the prosthesis plate to the tympanic membrane/malleus handle is missing. Larger rotations can be a cause for larger deviations between the 1-point and 5-point method.

### Comparison of Prosthesis types

The comparison between clip and bell-type prostheses demonstrated that both types generally provide good sound transmission, with only a small loss (less than 10 dB) in transmitted sound magnitude (see Fig. 3) and a THD of less than − 20 dB for equivalent 85 ± 15 dB SPL excitation (see Fig. 5) for all prosthesis types. The latter translates into a THD of < 1%, which is generally considered good to excellent sound quality [17]. This consistency across different types suggests that modern prosthesis designs are optimized for uniform acoustic performance across a wide frequency range [18].

Mean magnitude loss difference is not significant between prosthesis types. However, we did observe a notable exception in the 0.3–0.7 kHz band, where Type2 and Type3 + crimp showed a significant difference in magnitude loss.

This difference might be attributed to the additional crimping step in the Type3 + crimp, which enhances the stability of the coupling and possibly reduces sound loss at lower frequencies. However, the means in each band are influenced heavily by the relative location of the resonance peaks present in the individual data (see Fig. S1); the net negative loss (gain) for Type3 + crimp is a result of this. A larger database would be required for definite conclusions. Moreover, the unique design of the Type 3 prosthesis, which features a clip shape but functions as a bell prosthesis in clinical practice, makes the crimping procedure more challenging to control compared to other common bell-type prostheses. This increased difficulty could also contribute to the observed differences.

It must also be noted that while sound quality is excellent overall, all prostheses introduce some level of distortion, as evident from the THD measured across the coupling interface (Table 1). Some prosthesis designs (Type2, Type5) seem to be more prone to distortion than others. To the best of our knowledge, this is the first study measuring THD in ossicular replacement prostheses. From general knowledge about mechanical sound transmission systems such as loudspeakers [19], we can project that the distortion of the signal arises from imperfections in the coupling between prosthesis and ossicles, introducing mechanical resonances, damping, or friction at the coupling interface. This results in nonlinearities in the mechanical transmission of sound and creates harmonic overtones. This would suggest that the prostheses with less THD have a tighter coupling to the stapes head. In the living organism, tissue growth around the coupling might affect final THD as well.

Overall, our study found no systematic differences in sound transmission and distortion between clip and bell-type prostheses. This finding aligns with clinical observations suggesting that both clip and bell types perform postoperatively well in practice [20–24]. Comparative audiological study revealed only minor differences between the two prosthesis types [25]. The slight discrepancy between clinical and experimental results could be due to confounding factors present in clinical settings, such as the condition of the middle ear mucosa and aeration and prosthesis handling characteristics [15, 26–28].

### Evaluating coupling technique: Loose vs. tight crimping

As illustrated in Sect. 2.2, the Type3 prosthesis was coupled to the stapes head in two ways: loosely placed or tightly crimped. The results of THD change at the coupling interface (see Table 1) suggest that the crimped installation is more stable in the lower frequencies (500 Hz frequency band) than the non-crimped version. A study on coupling to the incus rather than the stapes also reported a small increase in sound transmission loss (within 8dB on average) for loose or no crimp compared to optimal tight crimping [11, 29]. We also found a significant difference in THD between crimped vs. uncrimped state in the 4 kHz band (see Table 1), although total THD is still very low at < 0.1% in both cases.

In general, the statistical results in Figs. 4 and 6 suggest that, in terms of sound quantity (magnitude loss at the coupling interface) and sound quality (overall THD on the stapes footplate), both loose and tight couplings are effective for the Type 3 prosthesis as long as they are properly connected.

### Other factors affecting outcome

Our results suggest that different coupling designs do not affect sound transmission. However, other factors need to be considered in terms of clinical outcome. Reducing postoperative dislocation is essential for the long-term success of ossicular chain reconstructions [30], which is greatly influenced by the coupling mechanism. Clip interfaces have been shown to be less prone to dislocation than bell types [2, 31]. Improved handling capabilities of the coupling elements enhance surgical success by making the prostheses easier to attach and adjust [9, 20]. Furthermore, ensuring geometrical conformity between the prosthesis and the stapes head is important for optimal hearing outcomes [32]. Advances in custom manufacturing, such as 3D printing, offer the potential for personalized fits, accommodating individual anatomical variations to improve coupling results [33, 34].

### 1-point vs. 5-point method

Our findings reveal that the choice of measurement method, 1-point or 5-points, significantly affects the statistical outcomes in reconstructed middle ear. However, the mean differences are < 5 dB for the whole frequency range. The results seem to suggest a smaller measurement error for Bell type prostheses when using a single point LDV than for Clip type prostheses, which might be an indication these are less prone to rotational or off-axis movement. This is possibly a result of a less rigid coupling to the stapes head for Clip type, allowing for rotation of the prosthesis around the stapes instead of translating these components to the structures further down. Note that TB models possibly lack some of the natural tension present in a living, complete ossicular chain due to the removal of partial structures for better visibility of the stapes. Conclusions drawn concerning performance in vivo may therefore not be completely accurate. Nevertheless, our findings indicate that the selection of appropriate measurement methods needs serious consideration for accurate acoustic assessment, such as the evaluation of prosthetic performance in reconstructed middle ear.

## Conclusion

All tested prostheses types performed uniformly good in terms of sound transmission. This means that the choice of a prosthesis in surgery can and must be based on other considerations, like e.g. middle ear pathology, handling during insertion, available proper prostheses lengths and so on, which finally influenced the postoperative coupling quality, and therefore hearing outcome. Using the measurement method proposed here allows for quantifiable and comparable assessment of the sound transmission at the coupling interface between the prosthesis and the stapes head, a critical factor in the success of partial ossicular chain reconstructions for conductive hearing loss. Further research might include a comprehensive comparison of different prosthesis types using both clinical data and an experimental setup similar to the one developed here.

## Electronic supplementary material

Below is the link to the electronic supplementary material.

Supplementary Materials (Fig.S1, Table S1-4)

## Data Availability

Data associated with the study has not been deposited into a publicly available repository and data will be made available on request.
