# Supplementary material for: Investigation of the coupling quality of partial prostheses at the stapes head
Source: Eur Arch Otorhinolaryngol. 2024 Nov 29;282(5):2301–12. doi: 10.1007/s00405-024-09105-x (PMC12055659; doi:10.1007/s00405-024-09105-x)
Supplement: Supplementary file 1 — Supplementary Materials (Fig.S1, Table S1-4) [file 405_2024_9105_MOESM4_ESM.docx]

# Appendix. Supplementary materials


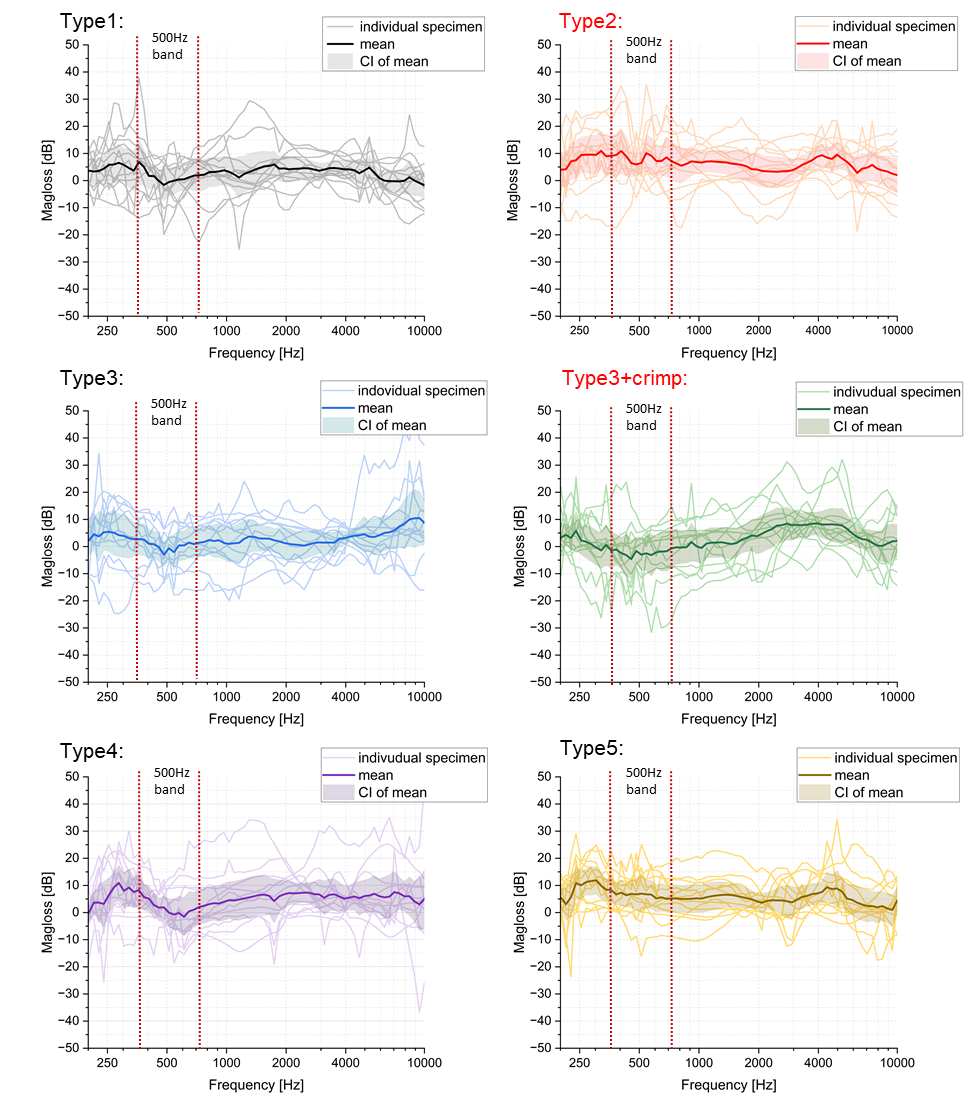


Fig. S1: Raw data of magnitude loss across the coupling interface at a range of frequencies (from 200 Hz to 10,000 Hz) for various types of clips and bells. (n=12)

| Frequency-Band | Lower Limit in Hz | Upper Limit in Hz |
| --- | --- | --- |
| 250 | 177 | 354 |
| 500 | 355 | 707 |
| 1000 | 708 | 1414 |
| 2000 | 1415 | 2828 |
| 4000 | 2829 | 5657 |
| 8000 | 5658 | 11314 |

Table S1. Frequency bands with equal logarithmic bandwidth. Limits are calculated based on geometric means between adjacent audiological frequencies. Each band covers the frequency from its lower limit to upper limit.

| Group | Band Frequency (k Hz) | 0.25  (0.177-0.354) | 0.5  (0.354-0.707) | 1  (0.707-1.414) | 2  (1.414-2.828) | 4  (2.828-5.657) | 8  (5.657-10) |
| --- | --- | --- | --- | --- | --- | --- | --- |
| Type1 | Mean  ± SD | -28,945  ± 8,990 | -34,719  ± 8,477 | -27,632  ± 9,579 | -26,970  ± 7,724 | -38,590  ± 7,946 | -60,313  ± 13,745 |
| Type2 | Mean  ± SD | -33,160  ± 8,983 | -39,424  ± 7,688 | -32,886  ± 9,975 | -28,843  ± 11,053 | -41,236  ± 7,689 | -66,484  ± 9,921 |
| Type3 | Mean  ± SD | -29,610  ± 9,798 | -33,232  ± 8,294 | -30,421  ± 10,540 | -35,301  ± 11,729 | -42,191  ± 12,507 | -62,627  ± 11,726 |
| Type3+crimp | Mean  ± SD | -25,950  ± 10,641 | -31,919  ± 9,658 | -30,290  ± 9,439 | -31,221  ± 6,949 | -42,747  ± 12,010 | -61,566  ± 11,081 |
| Type4 | Mean  ± SD | -29,808  ± 8,525 | -31,804  ± 7,469 | -28,222  ± 6,625 | -37,710  ± 8,090 | -43,856  ± 7,677 | -61,980  ± 10,646 |
| Type5 | Mean  ± SD | -30,834  ± 6,936 | -39,815  ± 6,490 | -36,981  ± 8,225 | -35,216  ± 10,870 | -43,870  ± 6,238 | -66,276  ± 6,316 |
| F Value | _ | 0.775 | 2.414 | 1.695 | 2.298 | 0.545 | 0.679 |
| P Value | _ | 0.571 | 0.045 | 0.148 | 0.055 | 0.741 | 0.641 |

Table S2: ANOVA test of total harmonic distortion at point c for prosthesis types examined. Significant values are marked in red color.

| Group | Group | Mean Difference | Std. Error | P value |
| --- | --- | --- | --- | --- |
| Type1 | Type2 | 4.70546 | 3.29538 | 0.710 |
|  | Type3 | -1.48742 | 3.29538 | 0.998 |
|  | Type4 | -2.91497 | 3.29538 | 0.949 |
|  | Type5 | 5.09573 | 3.29538 | 0.636 |
|  | Type3+crimp | -2.80026 | 3.29538 | 0.957 |
| Type2 | Type1 | -4.70546 | 3.29538 | 0.710 |
|  | Type3 | -6.19289 | 3.29538 | 0.424 |
|  | Type4 | -7.62043 | 3.29538 | 0.204 |
|  | Type5 | 0.39027 | 3.29538 | 1.000 |
|  | Type3+crimp | -7.50572 | 3.29538 | 0.218 |
| Type3 | Type1 | 1.48742 | 3.29538 | 0.998 |
|  | Type2 | 6.19289 | 3.29538 | 0.424 |
|  | Type4 | -1.42754 | 3.29538 | 0.998 |
|  | Type5 | 6.58315 | 3.29538 | 0.355 |
|  | Type3+crimp | -1.31284 | 3.29538 | 0.999 |
| Type4 | Type1 | 2.91497 | 3.29538 | 0.949 |
|  | Type2 | 7.62043 | 3.29538 | 0.204 |
|  | Type3 | 1.42754 | 3.29538 | 0.998 |
|  | Type5 | 8.01070 | 3.29538 | 0.161 |
|  | Type3+crimp | 0.11471 | 3.29538 | 1.000 |
| Type5 | Type1 | -5.09573 | 3.29538 | 0.636 |
|  | Type2 | -0.39027 | 3.29538 | 1.000 |
|  | Type3 | -6.58315 | 3.29538 | 0.355 |
|  | Type4 | -8.01070 | 3.29538 | 0.161 |
|  | Type3+crimp | -7.89599 | 3.29538 | 0.173 |
| Type3+crimp | Type1 | 2.80026 | 3.29538 | 0.957 |
|  | Type2 | 7.50572 | 3.29538 | 0.218 |
|  | Type3 | 1.31284 | 3.29538 | 0.999 |
|  | Type4 | -0.11471 | 3.29538 | 1.000 |
|  | Type5 | 7.89599 | 3.29538 | 0.173 |

*Table S3: Post-Hoc-Tests (Tukey HSD) of total harmonic distortion at point c for prosthesis types examined at frequency band 0.5 kHz. Significant values are marked in red color.*

| Group | Band Frequency (k Hz) | 0.25  (0.177-0.354) | 0.5  (0.354-0.707) | 1  (0.707-1.414) | 2  (1.414-2.828) | 4  (2.828-5.657) | 8  (5.657-10) |
| --- | --- | --- | --- | --- | --- | --- | --- |
| Type1 | Mean ± SD | -24,759  ± 7,819 | -29,494  ± 7,693 | -23,343  ± 8,086 | -23,113  ± 6,700 | -36,927  ± 6,362 | -62,416  ± 12,994 |
| Type2 | Mean ± SD | -21,192  ± 10,286 | -25,735  ± 10,787 | -24,039  ± 9,675 | -22,609  ± 8,378 | -33,733  ± 7,978 | -61,731  ± 8,830 |
| Type3 | Mean ± SD | -24,995  ± 7,424 | -25,034  ± 7,344 | -23,016  ± 7,682 | -29,306  ± 8,888 | -36,657  ± 8,566 | -53,964  ± 17,548 |
| Type3+crimp | Mean ± SD | -27,600  ± 8,401 | -30,731  ± 9,364 | -27,718  ± 9,610 | -29,148  ± 10,709 | -34,317  ± 10,945 | -57,572  ± 14,721 |
| Type4 | Mean ± SD | -25,232  ± 11,462 | -28,246  ± 8,484 | -23,850  ± 8,429 | -24,918  ± 6,194 | -37,309  ± 10,797 | -59,969  ± 9,182 |
| Type5 | Mean ± SD | -29,290  ± 7,075 | -29,835  ± 6,029 | -27,173  ± 8,015 | -29,723  ± 8,982 | -34,829  ± 9,950 | -61,023  ± 7,993 |
| F Value | _ | 0,756 | 0,909 | 0.676 | 1.830 | 0.323 | 0.783 |
| P Value | _ | 0,586 | 0,481 | 0.643 | 0.119 | 0.897 | 0.566 |

Table S4: ANOVA test of total harmonic distortion at point d for prosthesis types examined. Significant values are marked in red color.
